# The Relationship Between Adverse Childhood Experiences and Alzheimer's Disease: A Systematic Review

**DOI:** 10.3389/fnagi.2022.831378

**Published:** 2022-05-04

**Authors:** Kayla B. Corney, Emma C. West, Shae E. Quirk, Julie A. Pasco, Amanda L. Stuart, Behnaz Azimi Manavi, Bianca E. Kavanagh, Lana J. Williams

**Affiliations:** ^1^School of Medicine, IMPACT - Institute for Mental and Physical Health and Clinical Translation, Deakin University, Geelong, VIC, Australia; ^2^Institute of Clinical Medicine/Psychiatry, University of Eastern Finland, Kuopio, Finland; ^3^Barwon Health, Geelong, VIC, Australia; ^4^Department of Medicine-Western Health, The University of Melbourne, Melbourne, VIC, Australia

**Keywords:** Alzheimer's disease, dementia, cognitive aging, systematic review, adverse childhood experience (ACE)

## Abstract

**Background:**

Alzheimer's disease is a global health concern, and with no present cure, prevention is critical. Exposure to adverse childhood experiences may increase the risk of developing Alzheimer's disease. This systematic review was conducted to synthesize the evidence on the associations between adverse childhood experiences (<18 years) and the risk of Alzheimer's disease in adulthood.

**Methods:**

A search strategy was developed and conducted to identify articles investigating the associations between exposure to adverse childhood experiences and the onset of Alzheimer's disease by searching key databases (CINAHL, MEDLINE and PsycInfo). Two reviewers independently determined the eligibility of studies according to pre-determined criteria, and assessed the methodological quality using the US National Heart, Lung and Blood Institute 14-item checklist for observational cohort and cross-sectional studies, respectively. Due to limited studies, a descriptive synthesis was performed. The protocol for this review is published in BMJ Open and registered with PROSPERO (CRD42020191439).

**Results:**

Our search yielded 781 articles, of which three (two separate analyses from the same cohort study and one cross-sectional study) met the predetermined eligibility criteria. The methodological quality assessment yielded an overall mean score of 78.9% (range 66.6 – 84.6%). All studies found adverse childhood experiences were associated with an increased risk of Alzheimer's disease. However, there was a limited number of available studies to inform the synthesis.

**Conclusions:**

Adverse childhood experiences appear to be associated with an increased risk of Alzheimer's disease, although, further research is needed.

**Registration and Protocol:**

The protocol for this review is registered with PROSPERO (CRD42020191439) and published with BMJ Open (Corney et al., 2021).

## Introduction

Alzheimer's disease (AD) is an incurable neurodegenerative disorder and the most common form of dementia, affecting approximately 70% of people with the disease, and has a high personal and societal impact (Thies and Bleiler, 2013). Clinically, AD initially presents with episodic memory impairment, subsequently followed by executive dysfunction, confusion, agitation, sleep disturbance, language difficulties and behavioral changes (Blazer et al., 2015; Bahnasy et al., 2018; Lane et al., 2018). Although previous research has revealed many aspects of AD, it is yet to be known of the exact etiology, the disparity in disease progression, and how AD can be prevented or cured.

Recently, distinct pathological changes have been reported to be linked to AD, with the loss of proteostasis being the primary theory, specifically affecting the amyloid and tau proteins, which in turn, causes a cascade of detrimental events. These changes are followed by neuron death and the onset of AD (Kametani and Hasegawa, 2018). Additionally, the vascular dysregulation hypothesis has also been proposed, suggesting changes between the blood flow substrate pathway and neuron and glial energy levels from age dependent blood brain barrier permeability breakdown. This impacts on misfolded protein removal, and ultimately, detrimental effects correlating with cognitive disorders (Iadecola, 2013; Iturria-Medina et al., 2016). Previous evidence has reported vascular dysregulation as an initial biological event during cognitive decline, followed by amyloid deposition, metabolic dysregulation, physical impairment and brain atrophy (Iturria-Medina et al., 2016).

Genetics have also been reported to play a role in AD progression, with overexpression associated with increased amyloid burden. In early-onset AD, common genes include APP (genes encoding γ-secretase complex), presenelin-1 and presenelin-2 in chromosomes 21, 14 and 1, and in late-onset AD, apolipoprotein E series, especially APOE4, is the major genetic risk (Theendakara et al., 2016; Simic et al., 2017). However, despite this accumulating evidence, current identified factors do not explain the full extent of disease onset. Thus, the role of additional factors needs to be explored further. Similarly, epigenetic pathways have been assumed to play a key role in cognitive dysfunction and AD, suggesting early life stress such as ACEs are essential for brain functioning (Lemche, 2018). The epigenetic theory of latent early associated regulation proposed by Lahiri and Maloney (2010) posits that ACEs can change gene expression lasting long periods of time, and therefore, alterations of gene expression linked to cognitive disorders only becomes apparent later in life.

One such factor, may be exposure to adverse childhood experiences (ACEs), which refers to sources of trauma or stress occurring under the age of 18. ACEs include emotional, physical and sexual abuse, emotional and physical neglect, and exposure to household challenges, such as domestic violence, substance abuse, mental illness, criminal behavior and parental loss (death, separation and divorce) (Felitti and Maloney, 1998). In recent years, a growing body of research has highlighted the harmful effects of ACEs on health throughout the life span, including evidence of an association between ACEs and an increased risk for cognitive decline (Richards and Wadsworth, 2004; Ritchie et al., 2011; Korten et al., 2014) and AD (Norton et al., 2011, 2016; Radford et al., 2017). Additionally, ACEs may increase the risk of AD by amplifying the risks of other known factors in the development of AD, such as inflammation, depression and smoking (Felitti and Maloney, 1998; Chapman et al., 2004; Danese et al., 2009; Tani et al., 2020). Similarly, a higher exposure of ACEs has been found to disrupt normal psychological development, leading to an increased risk of negative health outcomes, which may in turn, have the potential to increase the risk of AD (Felitti and Maloney, 1998; Danese et al., 2009; Burke et al., 2017; Tani et al., 2020). Moreover, recent research has reported traumatic early life experiences can change stress regulatory functions, leading to later altered stress responses (Bellis, 2014; Fink and Galea, 2015). Increased stress levels are reported to increase amyloid burden, thus increasing cognitive decline prior to AD progression (Burke et al., 2017). Therefore, ACEs, in conjunction with factors that initiate a stress response, could impact the risk of AD.

Chronic stress throughout the adult lifespan has been consistently associated with a decline in cognitive function, and an increased risk of dementia. Despite this body of evidence linking life stress to dementia, few studies have examined the relationship between adverse events early in life and AD. Nevertheless, previous studies have reported a decline in cognition to begin years before clinical signs of AD (Shea and While, 2018). From this perspective, the risk factors must have occurred before this antecedent period, and thus, ACEs may be a potential factor influencing the onset of AD. In addition, previous evidence reports positive social factors such as social support, family history and social activities to be protective against AD (Bellis, 2014; Fink and Galea, 2015; Burke et al., 2017; Shea and While, 2018), which therefore suggests, in reverse conclusion, negative influences of ACEs.

### Objective

While adverse experiences throughout the lifespan have been reported to be a risk factor for cognitive decline, associations between adverse experiences in early life and AD specifically remain unclear. We aim to identify and synthesize the current literature to provide an indication of the current quality and level of evidence, and directions for future research on the association between ACEs (occurring before the age of 18 years) and the risk of AD in adulthood. Given the multi-factorial nature of AD, a better understanding of how adverse events throughout early life influence AD may help provide strategies to combat the growing burden of AD.

## Methods

The protocol for this systematic review was published (Corney et al., 2021) and registered with the International Prospective Register of Systematic Reviews (PROSPERO) **(**CRD42020191439). This systematic review was conducted according to the Preferred Reporting Items for Systematic Reviews and Meta-analyses (PRISMA) (Moher et al., 2009).

### Selection Criteria

#### Population

Worldwide, peer-reviewed studies published in English were eligible if they were longitudinal cohort, case-control and/or cross-sectional in design. Investigating participants from general and clinical populations who were exposed to any ACE before the age of 18 years and AD, with no other restrictions on participant demographics (e.g., sex/nationality).

#### Exposure

The exposure included any ACE before 18 years of age, defined by emotional/physical/sexual abuse, emotional/physical neglect, and household challenges, such as exposure to domestic/family/intimate violence, substance abuse, mental illness, criminal behavior and parental loss (death, separation and divorce) (Felitti and Maloney, 1998).

#### Comparison

Only studies with an appropriate comparison group, such as participants not exposed to any ACE were eligible.

#### Outcomes

For eligibility purposes, the outcome of a diagnosis of AD was consistent with an internationally recognized clinical or diagnostic classification system such as the International Classification of Diseases (ICD), Diagnostic and Statistical Manual of Mental Disorders (DSM), National Institute of Neurological and Communicative Diseases and Stroke/Alzheimer's Disease and Related Disorders Association (NINCDS-ADRDA criteria), and/or National Institute on Aging–Alzheimer's Association (NIA-AA workgroup criteria).

#### Exclusions

Studies were excluded if they were randomized controlled trials in design or published in a language other than English.

### Search Strategy and Information Sources

In consultation with an academic librarian, a search strategy was developed combining elements of the Population, Exposure, Comparator, and Outcomes (PECO) inclusion criteria, and translated for each research database (CINAHL Complete, MEDLINE Complete and PsycInfo) (Table 1). One reviewer (K.B.C.) implemented the initial search on the 11th of November 2020 for these databases *via* the EBSCOhost platform, a secondary search was performed on the 23rd of March 2022 to identify any new studies. Limitations were applied, restricting studies published in a language other than English. Gray literature was searched using an adapted search in Google. In addition, hand-searching the reference lists of included studies was performed to identify any further studies. A complete search strategy for each database is presented in Tables 1–**3**.

**Table 1:** Search terms.

(“Child*” OR Young* OR Early) AND (Physical* OR Emotion* OR Sexual*) AND (Abuse OR Neglect) OR (“Adverse childhood experiences” OR “Child abuse+” OR “Parental death+” OR “Child of impaired parents” OR “Divorce” OR “Domestic violence+”) OR (Parental crime OR Parental alcohol abuse OR Parental drug abuse) AND (“Alzheimer disease” OR “Dementia”)

#### Selection Process

Two reviewers (K.B.C. and E.C.W.) independently screened the titles/abstracts and reviewed full texts according to a predetermined screening checklist based through covidence data extraction and agreed 100% on selection. One reviewer (K.B.C.) screened the reference lists of eligible studies to locate any further articles.

### Data Collection and Effect Measures

#### Data Items

A data extraction form was developed in Covidence and reviewed by K.B.C and E.C.W in addition to the supervising author L.J.W. Next, pertinent data were extracted by K.B.C and E.C.W independently, including: citation/study details (i.e., author/study/year/country); study approach (i.e., aims/design/setting); participant/population information (i.e., age/sex/demographics/sample size); exposure information (i.e., number/type of ACEs/age of exposure); comparator information; outcomes (i.e., diagnosis of Alzheimer's disease); and key results.

#### Effect Measures

The effect measures for this review included odds ratio [95% confidence interval (95% CI) or *p* value], as well as hazards ratio and Wald statistic [degrees of freedom (*df* ), *p* value].

### Assessment and Reporting of Methodological Quality of Included Articles

The methodological quality was assessed using the US National Heart, Lung and Blood Institute 14-item checklist for observational cohort and cross-sectional studies, assessing study population, assessment of risk, analysis and data presentation, study design, and assessment of outcome (National Institutes of Health, 2021). The ratings were performed by K.B.C and E.C.W independently, and discrepancies were resolved through one consensus meeting. The overall quality of eligible studies was categorized using the predetermined criteria as follows: good, fair or poor, with a rating of poor translating to a high risk of bias.

### Synthesis of the Findings

A description of included studies and their methodological quality are presented in text and in Table 2. In addition, a descriptive synthesis of the key findings is presented in text, with key results and summaries of associations shown in Table 3. A small number of included studies and the nature of different methodological approaches precluded undertaking a meta-analysis, which was confirmed in consultation with a statistician.

**Table 2:** Descriptive characteristics of eligible studies included in this review in order of publication.

**First author (ref). title, year country**	**Study design**	**Year of data collection and/or follow up period**	***n* (% female)**	**Population description**	**ACE type/number**	**Age (years) mean (+ SD)**	**ACE – Identification method**	**AD – Identification method**
Norton et al. (2011) Early parental death and remarriage of widowed parents as risk factors for Alzheimer disease: The Cache County Study, USA.	Longitudinal Cohort	1995–1997, 1998–2000, 2002–2004, 2005–2007	4,108 (57.4%)	Participants were recruited from The Cache County Study on Memory Health and Aging. A longitudinal, population-based study of (AD) and other dementias. The study was initiated in 1995, with the goal to examine genetic and environmental risk factors of AD and other dementias. Eligible participants were residents of Cache County, a rural area located in north eastern Utah.	1–Parental death	75.7 (±7.1)	The Utah Population Database of linked population-based information.	Clinical diagnosis according to the National Institute of Neurological and Communicative Diseases and Stroke/Alzheimer's Disease and Related Disorders Association criteria for Alzheimer's disease (NINCDS-ADRDA).
Norton et al. (2016) Family member deaths across adulthood predict Alzheimer's Disease risk. The Cache County Study, USA.	Longitudinal Cohort	1995–1997, 1998–2000, 2002–2004, 2005–2007	4,545 (56.7%)	As above.	1–Familial death	75.0 (±6.9)	As above.	As above.
Radford et al. (2017) Childhood Stress and Adversity is Associated with Late-Life Dementia in Aboriginal Australians, Australia.	Cross-sectional	March 2010 until September 2012	296 (61.8%)	Participants were Aboriginal and Torres Strait Is- lander Australians, from urban and rural communities who took part in the Koori Growing Old Well Study (KGOWS).	1–Childhood Aversity pooled result	66.1 (± 5.8)	The short form of the Childhood Trauma Questionnaire and the Koori Growing Old Well Study life course survey.	Clinical diagnosis based on the National Institute of Aging/Alzheimer's disease Association (NIA-AA) workgroup criteria.

**Table 3:** Results of eligible studies included in this review pertaining to the association between ACEs and AD.

**First author (ref), country, year**	**Data analysis**	**Presented values**	**Quality rating**	**Results**	**Adjusted for confounders**	**Summary of associations**
Norton et al. (2011), USA.	Logistic regression analyses	Odds ratio (*p* value)	Good−11/13	Maternal & Paternal death - Overall: *p* = 0.005 0 – 4 years: 1.413 (0.243) 5 – 10 years: 0.736 (0.348) 11 – 17 years: 2.315 (0.001) Maternal death - Overall: *p* = 0.005 0 – 4 years: 1.452 (0.207) 5 – 10 years: 0.735 (0.346) 11 – 17 years: 2.266 (0.001) Paternal death - Overall: *p* = 0.073 0 – 4 years: 2.228 (0.009) 5 – 10 years: 1.145 (0.628) 11 – 17 years:1.059 (0.803) Adjusted for gender - Maternal & Paternal – 1.337 (0.005) Maternal - 1.338 (0.004) Paternal - 1.331 (0.005) Adjusted for e4 allele - Maternal & Paternal – 1 copy= 3.093 (<0.001)/2 copies = 10.558 (<0.001) Maternal – 1 copy = 1 copy= 3.083 (<0.001)/2 copies = 10.852 (<0.001) Paternal - 1 copy = 1 copy= 3.086 (<0.001)/2 copies = 10.558 (<0.001) Orphanhood – (OR = 1.59, Wald = 0.78, df = 1, *p* = 0.378) Parental age at participants birth - Maternal [OR = 1.02, (0.017)] / AD net [OR = 1.01, (0.403)] Paternal [OR = 1.01, (0.019)] / AD net [OR = 1.00, (.966)] Maternal death during the participants adolescence [OR = 2.27, (0.001)] Paternal death before age 5 [OR = 2.10, (0.021)] Major depressive disorder lifetime history - Maternal death during adolescence had a MDD prevalence of 30%, and subjects exposed at ages younger than 11 had a MDD prevalence of 23% (χ^2^ = 2.645, df = 2, *p* = 0.266).	APOE, education, age, gender, SES	Both maternal and paternal death together were associated with a higher rate of AD. Maternal death was significantly associated with higher odds of AD, with the risk more than doubling with maternal death occurring during adolescence (11 – 17 years) and paternal death before age 5. Furthermore, orphanhood was not associated with a higher risk of AD. Moreover, investigation of parental death with and without remarriage of a widowed parent during the remaining years of a participant's childhood, found paternal death was no longer associated with AD. Although, maternal death during adolescence was associated with a higher risk of AD when the widowed father did not remarry. Participant gender and e4 allele was tested as a potential moderator of maternal and paternal death and AD risk. Gender or e4 allele did not moderate either association. Additionally, both maternal age and paternal age at the participants birth were associated with a higher risk of AD. However, when tested for their association with AD net of maternal and paternal death, both maternal age and paternal age at the participants birth were no longer associated with AD. However, the associations between maternal death during the participants adolescence and paternal death before age 5 with AD remained robust after adjustment for parental ages at participants' birth. Furthermore, they also investigated a lifetime history of MDD, Participants with maternal death during adolescence had a MDD prevalence of 30%, and ages younger than 11 had a MDD prevalence of 23%. However, this association did not reach statistical significance.
Norton et al. (2016), USA	Cox regression analysis	Wald statistic, df, and p values	Good – 11/13	Aged 65–69 years - Wald = 5.79, df = 2, *p* = 0.055 Aged 70–74 years - Wald= 2.02, df = 2, *p* = 0.364 Aged 75–79 years - Wald= 0.63, df = 2, *p* = 0.729 Aged 80 years or older - Wald= 4.46, df = 2, *p* = 0.108 Cumulative deaths during childhood 65-69 years (Wald = 5.33, df = 2, *p* = 0.070) 70-74 years (Wald = 1.90, df = 2, *p* = 0.386) 75-79 years (Wald = 0.83, df = 2, *p* = 0.659) 80 years or older (Wald = 4.61, df= 2, *p* = 0.100)	APOE genotype, age, gender	There was a trend for the association between parental deaths and AD risk for persons aged 65–69 years. There was no association detected among persons aged 70–74 years, 75–79 years, and 80 years or older. Additionally, cumulative deaths during childhood were explored for each age group. The number of childhood deaths was not significant among all age groups.
Radford et al. (2017), Australia.	Multivariate logistic regression	Odds ratio (95% CI)	Fair – 8/12	1.77 (CI: 1.08–2.91)	Age	Higher adverse childhood experience scores from the Childhood Trauma Questionnaire-Short Form were associated with AD.

*AD, Alzheimer's disease; df, Degrees of freedom; CI, Confidence Interval; SES, Socioeconomic status; APOE, Apolipoprotein E; MDD, Major Depressive Disorder*.

## Results

### Study Selection

**Figure 1:**
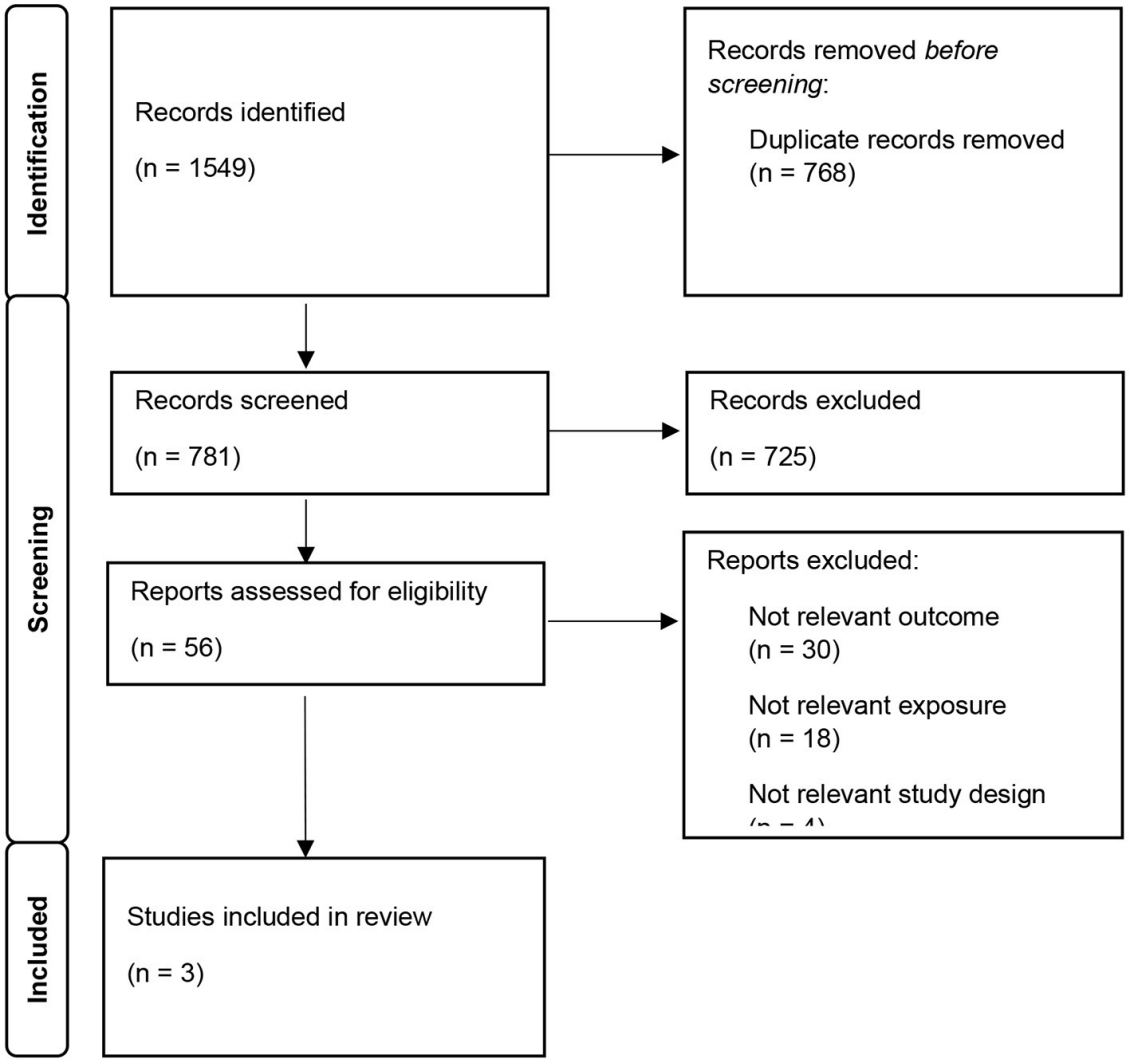
Prisma 2020 study screening flow chart and reasons for full-text screening study selection.

Figure 1 provides a flow diagram of the identified eligible articles (Page et al., 2021). Overall, the search of databases yielded a total of 1,549 articles, of which 768 were duplicates and were removed. Of the remaining 781 potentially relevant studies, 725 were excluded based on the titles/abstracts. Full texts of the remaining 56 articles were assessed against the pre-determined eligibility criteria. From this, 53 articles were excluded for the following reasons: 30 did not meet the outcome criteria, 18 did not meet the exposure criteria, 4 with ineligible study designs, and 1 further duplicate. No further articles were identified from the Google search or screening the reference lists. Thus, three articles were eligible for inclusion in this review. Two of the three articles identified utilized data from the same cohort, however, as a small number of articles were identified and they provided results for different outcomes, both studies were included and a descriptive synthesis was performed.

### Methodological Quality of Included Studies

The overall mean methodological quality score was 78.9% (range 66.6–84.6%) found in Table 3. The two assessors K.B.C and E.C.W achieved 97.3% agreement, and all discrepancies were resolved by the two reviewers in one consensus meeting.

### Description of the Studies

Table 2 presents the characteristics of the three included studies. There were two separate longitudinal analyses derived from the Cache County Study on Memory Health and Aging, published in 2011 (Norton et al., 2011) and 2016 (Norton et al., 2016). The study was initiated in 1995, with the goal to examine genetic and environmental risk factors of AD and other dementias. Eligible participants were residents of Cache County, a rural area in north eastern Utah. To assess ACEs, clinical data from the Cache County Study was linked with objective Utah Population Database (UPDB) data on family deaths. The UPDB contains over 14 million records representing over 7 million participants, providing multigenerational family histories (Norton et al., 2011, 2016). In addition, there was one cross-sectional study from Australia, published in 2017 (Radford et al., 2017), utilizing data from the Koori Growing Old Well Study. This study explored healthy aging and memory, as well as prevalence and incidence of age-related disease in urban Aboriginal populations in the state of New South Wales in Australia. Sample sizes ranged from *n* = 296 (Radford et al., 2017) to *n* = 4,545 (Norton et al., 2016) and studies were similar in terms of sex, with studies ranging from 56 to 62% female participants.

In terms of the exposure measures, Radford et al. (2017) pooled ACEs, which comprised an overall ACE score using the Childhood Trauma Questionnaire-Short Form (CTQ). The CTQ is a 28-item, self- reported questionnaire administered to identify traumatic childhood events, including five subscales of physical/emotional/sexual abuse and physical/emotional neglect. Whereas Norton et al. (2011) and Norton et al. (2016) investigated one type of ACE (familial death).

To assess outcomes, both longitudinal analyses (Norton et al., 2011, 2016) used a clinical diagnosis according to the NINCDS-ADRDA criteria to identify AD, while the cross-sectional study (Radford et al., 2017) used the NIA-AA workgroup criteria. All three studies performed different statistical analyses. Norton et al. (2011) conducted logistic regression, reporting odds ratio and *p* value, while Norton et al. (2016) conducted cox regression, reporting Wald statistic, degrees of freedom and *p* value. Finally, Radford et al. (2017) conducted multivariate logistic regression, reporting odds ratio with 95% confidence intervals (Table 3).

### Synthesis

Table 3 presents the main results from each study. In cross-sectional analyses, Radford et al. (2017) reported those with higher ACE scores had 1.8-fold higher odds of developing AD [OR 1.77 (1.08–2.91)] compared to those without AD. In contrast, the two longitudinal studies (Norton et al., 2011, 2016) reported on familial death, with Norton et al. (2011) reporting the strength of association between parental death and AD to depend on timing within childhood of the parent's death; the odds of developing AD more than doubled with maternal death occurring during adolescence [11–17 years, OR 2.3 (*p* = 0.001)] and paternal death occurring when participants were aged 0–4 years [OR 2.2 (*p* = 0.009)]. Norton et al. (2016) reported a trend for the association between familial death and AD risk for persons aged 65–69 years (Wald = 5.79, df = 2, *p* = 0.055), and no association among persons aged 70–74 years, 75–79 years, and 80 years or older. Norton et al. (2011) also explored both maternal and paternal deaths during childhood, reporting orphanhood was not associated with a higher risk of AD. Additionally, Norton et al. (2016) examined cumulative deaths during childhood, reporting the number of childhood deaths were not significant among all age groups.

In addition, Norton et al. (2011) tested e4 allele as a potential moderator of parental death and AD associations. Presence of the e4 allele did not moderate parental death and AD risk. Norton et al. (2011) investigated a lifetime history of major depressive disorder (MDD). Participants with maternal death during adolescence had a MDD prevalence of 30%, and participants exposed younger than 11 had a MDD prevalence of 23%, however, were not statistically significant.

#### Sex

Differences in sex were observed. Norton et al. (2011) tested sex as a potential moderator of parental death and AD associations. Sex did not moderate either association. Additionally, Norton et al. (2011) identified sex differences between maternal and paternal death and AD; the odds of developing AD more than doubled with maternal death occurring during adolescence, whereas the odds of developing AD more than doubled with paternal death during ages 0–4. Norton et al. (2011) also examined parental death with/without remarriage of a widowed parent during the remaining years of a participant's childhood, finding paternal death was no longer associated with AD. Although, maternal death during participants adolescence was associated with a higher risk of AD when the widowed father did not remarry.

#### Age

Differences in age were observed in two studies (Norton et al., 2011, 2016). Norton et al. (2011) reported experiencing maternal death was significantly associated with a higher rate of AD (*p* = 0.005), with the prevalence more than doubling with maternal death during subject's adolescence [OR 2.266 (*p* = 0.001)] (11–17 years). However, no association was found between maternal death and AD in subjects aged 0–4 years and 5–10 years. Whereas, an association between paternal death and AD was reported in subjects aged 0–4 years [OR 2.228 (*p* = 0.009)], but not for subjects aged 5–10 years and 11–17 years. Additionally, Norton et al. (2011) also explored older parental age at participants birth and risk of AD in adulthood. Both maternal age [OR = 1.02, (*p* = 0.017)] and paternal age [OR = 1.01, (*p* = 0.019)] at the participants birth were associated with a higher risk of AD. However, when tested for their association with AD net of maternal and paternal death, both maternal age and paternal age at the participants birth were no longer associated with AD. However, the associations between maternal death during the participants adolescence [OR = 2.27, (*p* = 0.001)] and paternal death before age 5 [OR = 2.10, (*p* = 0.021)] with AD remained robust after adjustment for parental ages at participants' birth.

Furthermore, Norton et al. (2016) reported a trend for moderation of familial deaths by age group (*p* = 0.092). When separate models were run for each of the four age groups, a trend was found for the association between familial deaths during childhood and AD risk for persons aged 65–69 years (Wald = 5.79, df = 2, *p* = 0.055), whereas no association was found among persons aged 70–74 years, 75–79 years, and 80 years or older.

## Discussion

This systematic review identified and evaluated existing research investigating the association between ACEs and AD. AD risk was reported to be higher for those with ACEs overall and for specific age groups and sex among these studies. However, it appears this topic remains substantially understudied, and the findings from this synthesis should be interpreted with caution.

While limited evidence was found, the identified literature suggests ACEs are associated with increases in AD risk. Suitably, there are a number of mechanisms which could explain these findings. Recent studies have noted age and sex differences can modify stress responses to ACEs, with these differences interacting with biological development, and therefore increasing the susceptibility to AD (Podcasy and Epperson, 2016). Previous research has found stress to be associated with AD risk, for example, high glucocorticoid levels have lasting effects that gradually increase neuron cell death (Norton et al., 2011). ACEs have been associated with dysfunctional neurodevelopment, as well as diminished development of the hypothalamic pituitary adrenal axis and stress responses (Radford et al., 2017). From this point of view, childhood is considered as a period of great development that may be sensitive to such adverse events. Subsequent chronic inflammation may precipitate the amyloid cascade, and therefore, accelerate the onset of AD (Radford et al., 2017). Similarly, ACEs have been reported to change stress regulatory functions, leading to altered stress responses later in life (Bellis, 2014; Fink and Galea, 2015). Increased stress levels are reported to increase amyloid burden, and thus increasing the risk of AD (Burke et al., 2017). Therefore, ACEs that initiate a stress response, could impact the risk of AD. Additionally, previous research has reported ACEs to be associated with many other negative health conditions, such as depression, anxiety, alcohol use and smoking, which have been found to be risk factors for AD (Felitti and Maloney, 1998; Chapman et al., 2004; Danese et al., 2009; Tani et al., 2020). Therefore, ACEs may also indirectly increase the risk of AD through other known risk factors.

Interestingly, the identified studies were mainly from rural populations. Norton et al. (2011) and Norton et al. (2016) utilized data from a rural area in the north eastern of Utah, and Radford et al. (2017) utilized data from an Aboriginal population from urban and regional communities in New South Wales, Australia. Previous evidence has reported people living in rural and remote locations generally experience poorer health outcomes compared to urban locations (Wakerman and Humphreys, 2011). Similarly, rural and urban differences in the prevalence of cognitive dysfunction have also been reported (Nunes et al., 2010; Russ et al., 2012; Nakamura et al., 2016). For example, Russ et al. (2012) provided evidence of a positive association between rural living and AD, identifying social isolation, education attainment and smoking are potential modifiable risk factors and possible interventions for poor cognitive health. Russ et al. also suggested that rural living, especially in early childhood, may affect the risk of developing AD. In this view, the current findings of ACEs increasing the risk of AD, may be due, at least in part, to the utilization of data from a high-risk population for poor cognitive health.

### Identification of Gaps in the Literature

Many questions remain unanswered regarding the relationship between ACEs and AD, due in part to the limitations in the literature, and the limited number of included studies. First, as mentioned above the identified studies were from rural populations, and thus, future studies expanding various populations, and investigating the influence of rural and urban differences are needed. Second, one of the three studies were cross sectional, with the remaining two studies utilizing the same study population, and thus, further longitudinal cohort studies are required to assess whether ACEs are a risk factor for AD. Third, all identified studies used a different exposure measure, such as one type of ACE or an overall score, which could potentially be predictors for the risk of AD, and therefore, future research should consider examining all types of ACEs. Fourth, the mechanisms of AD in individuals with a history of ACEs remains to be better characterized, and thus future research should consider mediation and moderation. Fifth, the current review was created in line with the current evidence on the definition of adverse childhood experiences (ACE) mentioned above, however future research could consider other adversities such as maltreatment and/or trauma and their associations with AD risk. Sixth, the relationship between ACEs and AD may vary by age, sex, number and/or type, however, the identified eligible studies only evaluated some of these relationships, and thus, future research should incorporate these factors.

### Strengths and Limitations

#### Strengths and Limitations of the Included Studies

A strength of all studies was employing an internationally recognized clinical diagnostic tool as their instrument of AD identification. Additionally, Norton et al. (2011) and Norton et al. (2016) were population-based prospective cohort studies. In contrast to case control studies, which identify cases and matched controls at the same time of disease, this study identifies a cohort of potential cases and controls prospectively before the time of disease in a population of persons of older age, a known risk factor for AD. Furthermore, Norton et al. (2011) and Norton et al. (2016) had a 90% response rate, which greatly reduces bias due to non-responders. Moreover, Norton et al. (2011) and Norton et al. (2016) linked data from the UPDB of linked population-based information, providing access to millions of individuals multigenerational family history (Norton et al., 2011, 2016).

In terms of limitations, one of the three studies was cross-sectional, which limits assumptions about causality, with a relatively smaller sample size (*n* = 296), compared to the two analyses from the cohort study that included a much larger sample size of *n* = 4,108 (Norton et al., 2011) and *n* = 4,545 (Norton et al., 2016). In addition, two (Norton et al., 2011, 2016) out of the three studies adjusted for sex, education and the apolipoprotein E genotype; only one study adjusted for MDD and socioeconomic status (SES) (Norton et al., 2011). Another notable factor is that two of the higher quality studies (Norton et al., 2011, 2016) have used the same dataset, the Cache County Study on Memory Health and Aging. A further consideration, is the use of the CTQ to measure ACEs in one study (Radford et al., 2017), as remembering prior knowledge of the event might have influenced the ACE score, resulting in recall bias.

#### Strengths and Limitations of This Review

The main strength of this study is the novel focus on ACEs under the age of 18 years and AD, and identification of current gaps in the literature. Furthermore, two reviewers independently screened the titles/abstracts and full-text articles and rated the methodology quality of studies. This enhances the robustness of article selection process and in producing this synthesis.

Limitations of this review must be acknowledged as well. As the focus was specifically on AD, studies involving participants with diagnoses of other dementias were excluded, which may have provided further insights. Furthermore, the depth and breadth of the synthesis on associations between ACEs and AD, was limited by the paucity of available studies. In addition, with significant variation within studies, performing a meta-analysis was not possible. Moreover, given only three studies were identified publication bias may be at play. This may be a result of unpublished non-significant or negative studies. Lastly, while the identified studies described the relationship between ACEs and AD, the studies were limited in the number and type of ACEs, as well as confounders. Hence, although ACEs were found to increase the risk of AD in the identified studies, the findings from the synthesis should be interpreted with caution.

## Conclusion

We conducted a systematic review of published literature investigating the association between ACEs (<18 years) and the risk of AD in adulthood and evaluated the methodological quality of the identified studies. To our knowledge, this is the first review to systematically investigate the association between ACEs (<18 years) and AD risk. All studies found ACEs were associated with an increased risk of AD. In this view, childhood mental health may be a possible avenue for early prevention strategies targeting Alzheimer's disease. However, the number of available studies were not sufficient to determine certainty in the levels of evidence. Therefore, further research is needed to understand the relationship between ACEs and AD. Moreover, the underlying mechanisms require further exploration.

## Data Availability Statement

The original contributions presented in the study are included in the article/supplementary material, further inquiries can be directed to the corresponding author.

## Author Contributions

KC, LW, and JP planned and designed the study. KC implemented the search strategy. KC and EW independently screened the titles and abstracts according to the predetermined screening checklist. Conflicts at the screening stage were resolved through discussion with LW who provided final judgement. Final inclusions were decided by full-text reading of the articles by KC and EW independently, and consensus with LW and KC analyzed and interpreted the data and reported on the present findings. AS, SQ, BK, and BM provided critical feedback throughout the study. All authors contributed to the final version of the manuscript.

## Funding

KC was supported by the Australian Rotary Health/Bing Taylor PhD Scholarship. LW was supported by a NHMRC Investigator grant (1174060). EW and BS were supported by a Deakin University Postgraduate Research Scholarship (DUPRS). The funding sources will have no role in the study design, data collection, data analysis, data interpretation, writing of the report or the decision to submit the paper for publication.

## Conflict of Interest

JP was employed by Barwon Health. The remaining authors declare that the research was conducted in the absence of any commercial or financial relationships that could be construed as a potential conflict of interest.

## Publisher's Note

All claims expressed in this article are solely those of the authors and do not necessarily represent those of their affiliated organizations, or those of the publisher, the editors and the reviewers. Any product that may be evaluated in this article, or claim that may be made by its manufacturer, is not guaranteed or endorsed by the publisher.
